# Supplementary material for: MetaRibo-Seq measures translation in microbiomes
Source: Nat Commun. 2020 Jun 29;11:3268. doi: 10.1038/s41467-020-17081-z (PMC7324362; doi:10.1038/s41467-020-17081-z)
Supplement: Supplementary file 10 — Supplementary Data 7 [file 41467_2020_17081_MOESM10_ESM.zip › File2/Confidence_VeryHigh_Taxonomy/30710_out.krona.html]

Javascript must be enabled to view this page.

members
magnitude
magnitudeUnassigned
count
unassigned
taxon
rank

30710\_out

10

2
9
superkingdom

phylum
1239
8

species
2
1897027

SRS065504\_contig\_number\_33885SRS146813\_contig\_number\_contig-100\_260.162729

class
186801
6

order
6
186802

family
186803
5

1898203

SRS015431\_contig\_number\_75690SRS019068\_contig\_number\_115851SRS020394\_contig\_number\_contig-100\_1380.86562SRS098073\_contig\_number\_contig-100\_1618.91729SRS1041140\_contig\_number\_contig-100\_2363.109945
5
species

1897048

SRS011302\_contig\_number\_25594
1
species

201174
1
phylum

1
1760
class

85004
1
order

31953
1
family

1678
1
genus

1897062

SRS149325\_contig\_number\_6622
1
species


SRS011302\_contig\_number\_30383
1
